# Methods and Instruments to Measure ICU Healthcare Professionals' Workload Related to Medical Technology—Protocol for a Scoping Review

**DOI:** 10.1111/nicc.70373

**Published:** 2026-02-07

**Authors:** Geertje J. C. van Limpt, Manon A. Molenaar, Faridi S. Jamaludin, Catharina J. van Oostveen, Frederique Paulus, Marcus J. Schultz, Peter van Vliet, Laura A. Buiteman‐Kruizinga

**Affiliations:** ^1^ Department of Intensive Care Amsterdam University Medical Centre Amsterdam the Netherlands; ^2^ Department of Intensive Care Haaglanden Medical Centre The Hague the Netherlands; ^3^ Research Support, Medical Library Amsterdam University Medical Centre Amsterdam the Netherlands; ^4^ Health Services Management & Organisation, Erasmus School of Health Policy & Management Erasmus University Rotterdam the Netherlands; ^5^ Clinical Department of Cardiothoracic Vascular Surgery Anaesthesia and Intensive Care Medicine Medical University Wien Vienna Austria; ^6^ Mahidol–Oxford Research Unit (MORU), Faculty of Tropical Medicine Mahidol University Bangkok Thailand; ^7^ Nuffield Department of Medicine University of Oxford Oxford UK; ^8^ Department of Intensive Care Reinier de Graaf Hospital Delft the Netherlands

**Keywords:** healthcare professionals, intensive care unit, medical technology, workload, workload measurement

## Abstract

**Background:**

Healthcare systems increasingly adopt medical technologies in direct patient care, particularly in highly technological environments like intensive care units (ICUs). While these technologies aim to enhance clinical outcomes, they can also introduce complexities that affect healthcare professionals' workload. Measuring workload related to the use of medical technology is crucial to ensure technologies support rather than hinder care delivery. Workload in this context encompasses temporal demands, subtask frequency and cognitive demands—distinct from scoring systems determining staffing ratios.

**Aim:**

To identify methods and instruments to measure ICU healthcare professionals' workload during direct patient care activities involving medical technology.

**Study Design:**

We will follow the Joanna Briggs Institute framework and Preferred Reporting Items for Systematic reviews and Meta‐Analyses extension for Scoping Reviews guidelines, using narrative synthesis to summarise findings. Electronic databases MEDLINE, EMBASE, PsycINFO, CINAHL, Cochrane Library, ISI Web of Science, the WHO International Clinical Trials Registry Platform and Google Scholar will be searched for studies published 2010–2025 reporting primary data. Exclusion criteria: paediatric population, editorials, letters and patient‐based scoring systems (e.g., Therapeutic Intervention Scoring System–76; Nursing Activities Score). Two reviewers will independently screen records and extract data using standardised forms. Reporting quality will be assessed using a self‐developed tool. Findings will be presented in a flowchart, tables and figures.

**Relevance to Clinical Practice:**

This review will provide a comprehensive overview of workload measurement methods during direct patient care activities involving medical technology in ICUs, serving as a practical resource for evaluating the workload impact of existing and emerging technologies.

**Review Registration:**

Open Science Framework, registered on 26th of September 2024 (registration DOI: 10.17605/OSF.IO/2A97J, https://osf.io/2a97j/)

## Impact Statement

- What is known about the topic
  ○ Measuring healthcare professionals' workload related to the use of medical technology in the ICU is crucial to ensure new and evolving technologies support rather than hinder effective care delivery.
  ○ Workload is a multidimensional concept and various workload measurement methods exist.
- What this paper adds
  ○ A protocol to identify workload measurement methods and instruments from the ICU healthcare professional's perspective during direct patient care activities involving medical technology.
  ○ A protocol for a categorisation of workload measurement methods structured by workload domain (temporal, subtask frequency, performance‐, subjective‐ or psychophysiological‐based), type of task and type of medical technology.

**Abbreviations**
ICU: intensive care unit
JBI: Joanna Brigs Institute
MMAT: Mixed Methods Appraisal Tool
NAS: Nursing Activities Score
OSF: Open Science Framework
PCC: Population, Concept, Context
PRISMA‐ScR: Preferred Reporting Items for Systematic reviews and Meta‐Analyses extension for Scoping Reviews
TISS‐76: Therapeutic Intervention Scoring System–76

## 1 Introduction

Medical technologies used in direct patient care are rapidly evolving, particularly in highly technological environments such as intensive care units (ICUs) [1]. These technologies—both new and conventional devices for bedside clinical interventions and monitoring, such as mechanical ventilators and patient monitors—are becoming increasingly complex with expanding features, capabilities and automation. While they aim to improve clinical outcomes and reduce costs [2], they can also introduce new operational complexities that affect cognitive demands, temporal requirements and workflow patterns [3]. Understanding and measuring healthcare professionals' workload related to the use of medical technology is crucial to ensure new and evolving technologies support rather than hinder effective care delivery, particularly amidst current staff shortages and the already high mental workload in the ICU [4, 5].

Workload is a multifaceted concept with widely varying definitions in the literature [6]. In this context workload encompasses three components: temporal demands (time to complete a task), subtask frequency (rate of actions within a task) and cognitive demands (mental workload). Various methods exist to measure workload across multiple dimensions, including observational measurements, subjective assessments, psychophysiological measures and performance metrics [7, 8]. We specifically focus on workload experienced by healthcare professionals (HCPs) directly involved in patient care, including nurses, physicians, respiratory therapists and other clinical staff who operate medical technologies.

Scoring systems such as the Therapeutic Intervention Scoring System–76 (TISS–76) and Nursing Activities Score (NAS) assign fixed scores to standardised patient care activities to determine optimal nurse‐to‐patient staffing ratios in the ICU [9, 10]. While useful for staffing decisions, these scores cannot measure workload variability—for instance, how workload differs between ventilator types, provider expertise or training levels and are therefore excluded from this review.

Given the variety in measurement approaches and the increasing complexity of ICU medical technology, a comprehensive overview of available workload measurement methods for ICU settings is needed to guide future research and implementation.

### 1.1 Aim

The aim of this scoping review is to identify and evaluate methods and instruments to measure ICU healthcare professionals' workload during direct patient care activities involving medical technology.

## 2 Design and Methods

### 2.1 Design

We will follow the methodological framework outlined by the Joanna Briggs Institute (JBI) [11, 12]. The results will be reported according to the Preferred Reporting Items for Systematic reviews and Meta‐Analyses extension for Scoping Reviews (PRISMA‐ScR) [13]. In accordance with the PRISMA‐ScR guidelines, this scoping review is registered in the Open Science Framework (OSF) on the 26th of September 2024 (Registration DOI: 10.17605/OSF.IO/2A97J).

### 2.2 Research Questions

The primary research question is as follows:

- Which methods and instruments are used to measure ICU healthcare professionals' workload during direct patient care activities, and which have been applied to medical technology‐related tasks?

The secondary research question is as follows:

- What is the evidence for the feasibility, validity and reliability of these methods and instruments?

### 2.3 Eligibility Criteria

The eligibility criteria for this scoping review are based on the Population, Concept, and Context (PCC) mnemonic [12], as outlined in Table 1. We focus specifically on workload related to direct patient care activities involving medical technologies in the ICU.

**TABLE 1:** Eligibility criteria.

	Inclusion criteria	Exclusion criteria
Population (P)	Healthcare professionals directly involved in patient care (e.g., nurses, physicians, physician assistants, paramedics, respiratory therapists and ventilation practitioners)	– Volunteers and informal caregivers – Healthcare professionals not directly involved in patient care (e.g., pharmacists, radiologists, laboratory technicians)
Concept (C)	Methods and instruments to measure workload during direct patient care generally, and specifically during medical technology‐related tasks.	– Patient‐based scoring systems that determine nurse‐to‐patient ratios – Workload exclusively related to electronic health records
Context (C)	– All types of ICUs, including medical, surgical, neurological, cardiac, respiratory and mixed ICUs – Simulated ICU settings	– Paediatric or Neonatal ICUs – Step‐down or intermediate care units (e.g., rehabilitation and stroke units)
Study designs	– Studies with original primary data (including quantitative and qualitative study designs and pilot studies) – Trial registrations – Protocols	– Letters – Guidelines – Commentaries – Posters
Publication period	2010–2025	
Language	English, Dutch, German	

Additionally, we will include all types of study designs reporting original primary data, as well as trial registrations and protocols (see Table 1 for detailed inclusion and exclusion criteria). We will restrict our search to studies between 2010 and 2025 in order to focus on contemporary ICU technologies, as technological development in critical care has accelerated rapidly in recent decades.

### 2.4 Definitions

Workload in this context is defined as a multidimensional construct [6] encompassing three components: (1) temporal demand; the time required to complete a task, (2) subtask frequency; the rate of steps or actions within a task (e.g., the frequency of manual ventilator adjustments within the task ventilator management), and (3) cognitive demand; the interaction between the task and the healthcare professionals' cognitive abilities, that is, mental workload. Cognitive demand can be measured through performance‐based, subjective (self‐reported) or psychophysiological methods [7].

This definition allows us to identify workload measurement instruments and methods that capture not only the cognitive effort but also the objective, practical demands of a specific (technology‐related) task. Importantly, this perspective differs fundamentally from patient‐based scoring systems that measure nursing workload in terms of the volume of patient care activities, such as the TISS–76 and NAS [9, 10]. These scoring systems are designed from a patient perspective (‘how much care does this patient require?’), generating a sum score to determine patient–nurse ratios. While these systems include technological interventions in their scoring items (e.g., mechanical ventilation, haemodynamic monitoring), they assign fixed, standardised scores to each intervention regardless of the specific technology used, how the task is performed, the providers' expertise or organisational context. While useful for staffing decisions, they cannot isolate the workload of specific tasks or technologies, nor measure workload as defined in this review. Our review instead adopts the individual healthcare professional's perspective, focusing on instruments capable of measuring task‐ and technology‐specific workload variations (e.g., comparing the workload of different ventilators). Therefore, traditional patient‐based scoring systems are excluded from this review.

We define medical technology as any technological innovation, device or system that directly supports bedside clinical interventions and patient monitoring in the ICU. This includes devices primarily operated by healthcare professionals involved in direct patient care, such as mechanical ventilators, infusion pumps, continuous renal replacement therapy (CRRT) devices and patient monitors [14]. Studies focusing exclusively on electronic health records‐related activities (e.g., documentation, administration) without direct relation to direct patient care activities or medical technology use are excluded.

### 2.5 Search Strategy

Together with a qualified medical librarian (FJ), the search strategy will be developed using medical subject heading (MeSH) and text words related to our pre‐specified inclusion and exclusion criteria. We will conduct a search within the following electronic databases: MEDLINE, EMBASE, PsycINFO (all OVID), CINAHL (Ebscohost), Cochrane library (Wiley), ISI Web of Science (Clarivate), the WHO International Clinical Trials Registry Platform search portal (WHO ICTRP) and additionally Google Scholar. We will screen the reference lists of reviews and included studies for additional studies. The full search strategy for MEDLINE (OVID) is provided in Table S1.

Our search strategy will focus on methods for measuring workload during direct patient care, without initially limiting ourselves to medical technology. This approach allows us to address both components of our primary research question: we will first identify and describe all workload measurement instruments used in direct patient care in the ICU, then analyse which of these have been applied specifically to medical technology‐related tasks. We chose this strategy for two reasons: first, to provide a comprehensive overview of the broader landscape of workload measurements in ICU patient care; second, because workload measurement instruments used in non‐technology contexts may also be applicable to technology‐related tasks, and we aim to identify all such potential instruments. Moreover, adding technology‐specific terms would risk excluding relevant instruments that measure workload during technology use without explicitly stating ‘technology’ in searchable fields.

### 2.6 Study Selection

Search results will be imported into EndNote X9.3.3 (Clarivate Analytics, PA, USA) and de‐duplicated with DedupEndNote [15]. Two reviewers (GL and MM) will conduct the selection process according to the PRISMA‐ScR guidelines, using the Rayyan web application, a recommended screening tool known for its high usability and accuracy [16]. The reviewers will independently assess titles and abstracts using the eligibility criteria. To ensure consistency, a sample of 30 publications will be evaluated together, and eligibility will be discussed before screening all abstracts.

Full texts of studies identified as potentially eligible by both reviewers will be independently reviewed to confirm selection. In any case of disagreement, a discussion between the two reviewers GL and MM will be attempted first. If this is inconclusive, LBK will serve as a third reviewer. The selection process (identification, screening and inclusion of articles) will be presented in a flowchart according to the PRISMA‐ScR checklist, see Figure S1.

### 2.7 Data Charting Process

The research team has developed a data charting form in Microsoft Excel based on the JBI standardised data extraction form to collect the relevant information. The form will be piloted by two reviewers (GL and MM) on five articles to ensure consistency across reviewers and to revise the form if necessary. Each reviewer will extract the data from the included studies independently. Extracted data will include study demographics (author, year of publication, country of origin); study aim; study design; setting; population and sample size; type of task and type of medical technology; and a description of the workload measurement method or instrument. Categories for tasks, medical technologies and measurement methods will be developed inductively during the data synthesis phase, after completing data extraction, to ensure they align with the actual data from included studies.

We will also extract feasibility data (e.g., required training, frequency and duration of measurements), validity and reliability of the identified measurement methods. Given the variability in workload definitions in literature, we will extract how workload was defined and which domain of workload was investigated. We will categorise the measurement methods into five workload domains as introduced in the definition of workload: temporal, subtask frequency, subjective, psychophysiological or performance‐based; the latter three representing different measurement approaches for cognitive demand. One reviewer (GL) will be responsible for contacting the corresponding lead author of an included study when clarification or additional data are needed, with a maximum contact limit of three emails.

### 2.8 Assessment of Methodological Quality of Individual Studies

While methodological quality assessment is not mandatory in scoping reviews, we will assess the reporting quality of the identified measurement instruments using a seven‐item appraisal tool, developed by the research team (GL, CO and FP) based on the Mixed Methods Appraisal Tool (MMAT) [17], see File S1. The checklist will assess reporting on aspects such as feasibility, validity, reliability and contextual application, with detailed criteria and examples of adequate reporting for each item. This assessment will indicate the completeness of reporting and provide insight into the potential usefulness of the identified instruments in the ICU setting. Two authors (GL and MM) will independently assess the reporting quality of the identified instruments and methods.

### 2.9 Data Analysis and Presentation

Study selection will be presented using a PRISMA flowchart [13]. Two reviewers (GL and MM) will independently categorise instruments by workload domain, measurement method, type of task and type of medical technology, with disagreements resolved through discussion with a third reviewer (LBK). Workload domains include the following: temporal, subtask frequency, performance‐, subjective‐ or psychophysiological‐based.

We will perform a narrative synthesis by comparing methods within and across categories to identify similarities, differences and gaps in workload measurement approaches. The research team will meet regularly to discuss emerging patterns and reach consensus on categories and interpretation. Descriptive statistics will summarise instrument characteristics (e.g., frequency of use by domain, technology type and geographical distribution). Findings will be presented in tables and figures addressing each research question.

## 3 Discussion

As healthcare increasingly adopts technological advancements, evaluating workload is essential to understand their impact and develop strategies for effective integration into patient care. This scoping review will provide a comprehensive overview of the instruments and methods used to assess the workload of healthcare professionals in ICUs, particularly in the context of direct patient care involving medical technology.

This review has several strengths. First, we will use a rigorous and systematic search strategy, developed in collaboration with a qualified medical librarian, enhancing the comprehensiveness of our search. Second, we will apply a broader definition of workload encompassing both task load and mental workload in relation to specific tasks, ensuring inclusion of diverse measurement approaches. Third, although quality assessment is not mandatory in scoping reviews, we will assess reporting quality to provide transparency about the extent in which instruments are adequately described in the literature, enabling readers to judge their potential applicability.

### 3.1 Limitations

This review has several limitations. A key challenge is the conceptual heterogeneity surrounding ‘workload’. Terms such as task load, mental workload, cognitive load, mental capacity and effort are often used interchangeably in the literature. In most studies, researchers focus on measuring the amount of work, or ‘task load’, likely because it is more quantifiable and easier to assess. However, workload also includes cognitive and emotional demands placed on an individual, often referred to as ‘mental workload’, which is influenced by task complexity, environment and personal capacity—especially in the context of healthcare professionals. These concepts are distinct but often confused or used interchangeably with terms like cognitive load, mental capacity, effort and stress, leading to inconsistencies. This terminological heterogeneity may limit the comprehensiveness of our search, as relevant studies with alternative terminology could be missed despite our broad search strategy. Additionally, our restriction to English, Dutch and German publications may introduce geographic bias by excluding potential relevant studies in other languages. Finally, consistent with scoping review methodology, we will not exclude studies based on study quality, resulting in methodological heterogeneity.

### 3.2 Implications for Practice

This review will serve as a practical resource to guide instrument selection for measuring ICU workload related to existing and emerging technologies. By categorising instruments across workload domains, types of tasks and medical technology and evaluating reporting quality, researchers, nurses and clinicians can identify appropriate measurement approaches for their specific context. This will support evidence‐informed decisions about technology implementation that balance clinical benefits with healthcare professional wellbeing and sustainable work environments.

## 4 Conclusion

Multidimensional workload assessment from the healthcare professional's perspective is essential for understanding the impact of medical ICU technologies. This scoping review will probably be the first systematic overview of available measurement instruments and methods, categorised by workload domain and evaluated for reporting quality.

## Author Contributions

The study was conceptualised and designed by G.J.C.v.L., M.A.M., P.v.V., M.J.S., F.P. and L.A.B.‐K. The search strategy was developed by G.J.C.v.L., M.A.M. and F.S.J. G.J.C.v.L. and M.A.M. initially drafted the protocol, which was later revised by C.J.v.O., F.S.J., P.v.V., M.J.S., F.P. and L.A.B.‐K. All authors carefully reviewed the final protocol and provided their approval.

## Funding

The authors have nothing to report.

## Ethics Statement

The authors have nothing to report.

## Consent

The authors have nothing to report.

## Conflicts of Interest

The authors declare no conflicts of interest.

## Supporting information


**Figure S1:** PRISMA flowchart.

**File S1:** Appraisal tool to assess the reporting of workload measurement methods.

**Table S1:** Search strategy for MEDLINE (OVID).

## Data Availability

The data that support the findings of this study are available from the corresponding author upon reasonable request.
