# Supplementary figures and images for: Methods and Instruments to Measure ICU Healthcare Professionals' Workload Related to Medical Technology—Protocol for a Scoping Review
Source: Nurs Crit Care. 2026 Feb 7;31(2):e70373. doi: 10.1111/nicc.70373 (PMC12883006; doi:10.1111/nicc.70373)

**Figure S1.** PRISMA Flowchart

*
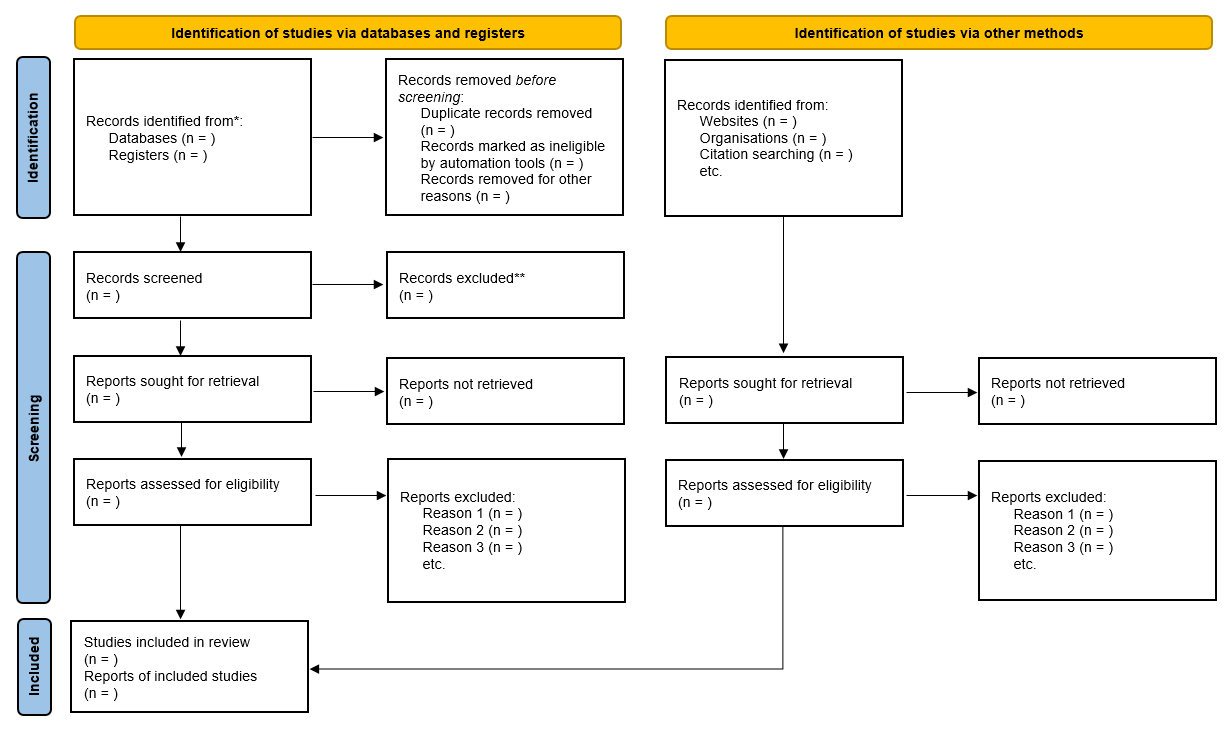
*

Supplement: Supplementary file 1 — Figure S1: PRISMA flowchart. [file NICC-31-0-s002.docx]
