# Supplementary material for: Methods and Instruments to Measure ICU Healthcare Professionals' Workload Related to Medical Technology—Protocol for a Scoping Review
Source: Nurs Crit Care. 2026 Feb 7;31(2):e70373. doi: 10.1111/nicc.70373 (PMC12883006; doi:10.1111/nicc.70373)
Supplement: Supplementary file 2 — File S1: Appraisal tool to assess the reporting of workload measurement methods. [file NICC-31-0-s001.docx]

**Supplementary File S1.** Appraisal tool to assess the reporting of workload measurement methods

|  | **Category** | **Criteria** | **Indicators of criteria not met** | **Yes / No** |
| --- | --- | --- | --- | --- |
| **1** | **Population** | ***Is the population clearly described?*** The target population should be explicitly identified and described. This includes demographic characteristics, professional roles, experience levels, work environment.   - "Nurses with minimum 2 years’ experience" - "Resident physicians in level-1 trauma hospital" | - Outcome of interest is a secondary outcome with different population than primary outcome, but this population is not described - Vague descriptions like "hospital staff" without specific details of profession |  |
| **2** | **Identification** | ***Is/are the measurement tool(s) clearly identified?***  The tool(a) should be explicitly named or sufficiently described, including its format and administration method. For established tools, the version information and original developers are described.  Examples;   - NASA Task Load Index (NASA-TLX) by Hart & Staveland (1988), administered digitally via tablet - A custom-designed cognitive load questionnaire with 12 items using 7-point Likert scales, administered on paper immediately after each task - Paper-based observation checklist with binary yes/no responses (see Figure 1 for full instrument) - Real-time physiological monitoring using Empatica E4 wristband | - Vague generic descriptions: "questionnaire", "rating scale", "data charting form", "assessment tool" without further explanation of content or structure - No description of format (paper/digital/app) or administration method - Modified tools without crediting original source - Multiple tools mentioned without clearly specifying which was used for the outcome of interest |  |
| **3** | **Purpose and construct** | ***Is the purpose and construct of the tool(s) clearly described?***  All tools intended use and the specific construct (e.g. subjective workload, time, task demand) are explained.   - "NASA-TLX measures subjective workload " - "Designed to measure time and task load (number of tasks per hour)" | - Vague descriptions like "measures workload" without further specification - No explanation of what aspects of workload are captured - Missing distinction between different types of workload (cognitive, temporal, task count) |  |
| **4** | **Validity** | ***Is there evidence or reference to the validity of the tool(s)?*** Information on how the validity was assessed. Content, construct, or criterion validity is addressed. | - No information or references about validation - Validity "assumed" but not tested or referenced - Validation evidence from totally different context without justification for generalizability |  |
| **5** | **Reliability** | ***Is there evidence or reference to the reliability of the tool?***    For instance test-retest reliability or consistency over time or across different observers. | - No information or references about reliability - Claims about tool quality without reliability evidence - Reliability "assumed" but not tested or referenced |  |
| **6** | **Feasibility** | ***Is/are the tool’s feasibility in the context discussed?***  The practicalities of using or implementing the tool in an specific environment, such as time, cost, technical requirements, and ease of use. Example:   - “10 minute administration time” - “to minimize disruption to patient care” - “No specialized equipment or training required” | - No information about practical implementation aspects - Feasibility "assumed" but not addressed |  |
| **7** | **Contextual application** | ***Is the contextual application of the tool(s) discussed?***  The specific context or activity in which the tool is applied should be described. Examples:   - "measured during medication administration tasks" - "management of tension pneumothorax" - "measured during in-hospital resuscitation" | - No identification of specific work context, tasks or activities - Generic descriptions like "during a shift", or "during work" without specifying concrete tasks or procedures - Unclear what aspects of work are being assessed |  |
