# Supplementary material for: Methods and Instruments to Measure ICU Healthcare Professionals' Workload Related to Medical Technology—Protocol for a Scoping Review
Source: Nurs Crit Care. 2026 Feb 7;31(2):e70373. doi: 10.1111/nicc.70373 (PMC12883006; doi:10.1111/nicc.70373)
Supplement: Supplementary file 3 — Table S1: Search strategy for MEDLINE (OVID). [file NICC-31-0-s003.docx]

**Table S1.** Search Strategy for MEDLINE (OVID)

| # | Searches | Results |
| --- | --- | --- |
| 1 | exp Medical Staff, Hospital/ or exp Physicians/ or exp Nurses/ or Nursing Staff, Hospital/ or exp Patient Care Team/ or nursing assistants/ or physician assistants/ or exp anesthetists/ | 419434 |
| 2 | (nurs* or perfusionist* or medical staff* or physician* or doctor* or medical specialist* or intensivist*).ti,ab,kf. | 1146013 |
| 3 | ((respiratory or respiration* or ventilation*) and (therapist* or practitioner*)).ti,ab,kf. | 7068 |
| 4 | 1 or 2 or 3 | 1350943 |
| 5 | exp Intensive Care Units/ or Critical Care/ or Critical Care Nursing/ | 160956 |
| 6 | (intensive care or critical care or ICU*).ti,ab,kf. | 277057 |
| 7 | 5 or 6 | 324744 |
| 8 | Workload/ or Work Performance/ or exp Occupational Stress/ or emotional exhaustion/ or subjective stress/ or time pressure/ or Time Management/ or exp "Task Performance and Analysis"/ or "Time and Motion Studies"/ | 85644 |
| 9 | (workload* or work load* or work volume* or work capacit* or task load* or cognitive load* or mental load* or mental pressur* or cognitive pressur* or task failure* or usability).ti,ab,kf. | 80618 |
| 10 | ((burden or stress or pressure or exhaustion or performance) adj3 (professional* or practitioner* or nurs* or doctor* or staff* or physician* or therapist*)).ti,ab,kf. | 19170 |
| 11 | 8 or 9 or 10 | 170673 |
| 12 | 4 and 7 and 11 | 3801 |
| 13 | (exp Child/ or exp Infant/) not exp Adult/ | 1972898 |
| 14 | exp Pediatrics/ or exp Intensive Care Units, Pediatric/ or neonatal intensive care.ti. | 94299 |
| 15 | ((child* or pediatric* or paediatric* or infant* or neonat*) not adult*).ti,ab,kf. | 2167569 |
| 16 | 13 or 14 or 15 | 2811268 |
| 17 | 12 not 16 | 3102 |
| 18 | exp Congresses as Topic/ or comment/ or editorial/ or (conference abstract* or poster presentation or trial registration).ti,ab,kf. or (comment* or editorial).ti. | 1748648 |
| 19 | 17 not 18 | 2990 |
| 20 | limit 19 to yr="2010 -2025" | 2621 |
